# Bibliometric and visual analysis of SGLT2 inhibitors in cardiovascular diseases

**DOI:** 10.3389/fphar.2024.1437760

**Published:** 2024-10-30

**Authors:** Runfang Pan, Yuqing He, Wan Melisandre, Yunyi Zhang, Wenyuan Su, Jiaming Feng, Chengyao Jia, Shaoling Li, Baonian Liu

**Affiliations:** ^1^ Department of Anatomy, School of Chinese Integrative Medicine, Shanghai University of Traditional Chinese Medicine, Shanghai, China; ^2^ Sport Medicine and Rehabilitation Center, Shanghai University of Sport, Shanghai, China; ^3^ Guanghua School of Stomatology, Sun Yat-Sen University, Guangzhou, China; ^4^ Department of Pathology, Shanghai Pulmonary Hospital, School of Medicine, Tongji University, Shanghai, China

**Keywords:** sodium-glucose transporter 2 inhibitor, cardiovascular disease, bibliometric study, Citespace, VOSviewer

## Abstract

**Background:**

Cardiovascular diseases (CVD) pose a significant threat to human health due to their high mortality and morbidity rates. Despite advances in treatments, the prevalence and impact of cardiovascular disease continue to increase. Sodium-glucose transporter 2 inhibitors (SGLT2i), initially approved for the treatment of type 2 diabetes, have important research value and promising applications in reducing CVD risk, especially in heart failure (HF) and atherosclerosis patients with cardiovascular disease (ASCVD). This study aims to comprehensively review the latest progress, research trends, cutting-edge hot spots, and future development directions of SGLT2i in the field of CVD through bibliometric analysis.

**Methods:**

Articles related to MSCs in cardiovascular diseases were sourced from the Web of Science. The bibliometric analysis was conducted using CiteSpace and VOSviewer, and a knowledge map was created based on the data obtained from the retrieved articles.

**Results:**

In this article, we screened 3,476 relevant studies, including 2,293 articles and 1,183 reviews. The analysis found that the number of papers related to the application of SGLT2i in CVD has generally increased, peaking in 2022. The United States and China contributed the largest number of papers, with the United States accounting for 36.97% of the total and also ranking first in terms of the number of citations. However, China’s high-quality papers are slightly lacking and need further improvement. Keyword analysis showed that empagliflozin, dapagliflozin, diabetes, and heart failure were the most common terms, reflecting the main research interests in currently published papers in this field.

**Conclusion:**

Bibliometric analysis showed a robust and growing interest in the application of SGLT2i for treating CVD. By summarizing the latest progress of SGLT2i in the field of CVD, exploring research hotspots, and looking forward to future research development trends, this article provides valuable insights for thinking about research prospects.

## 1 Introduction

Cardiovascular disease (CVD), a manifestation of systemic vascular disease or vascular disease in the heart and brain, is a serious threat to human health (Teo and Rafiq, 2021) because of its high mortality, high disability rate, high recurrence rate, and many complications (Roth et al., 2017). Although researches aimed at improving the health of patients with CVD have made progress, clinical morbidity, and mortality are still increasing (Damen et al., 2016; Wong and Sattar, 2023). Therefore, exploring new methods for treating CVDs has attracted wide attention. In recent years, the research on the role of SGLT2i in CVDs has always been a hot issue.

SGLT is widely present in the human body and has two main isoforms: SGLT1 and SGLT2. SGLT1 is expressed in the intestine, heart, and kidney, while SGLT2 is expressed almost exclusively in the kidney, distributed on the luminal side cell membrane of the proximal renal tubule, and is responsible for 90% of glucose reabsorption in the renal tubule (Chrysopoulou and Rinschen, 2024). Sodium-glucose cotransporter 2 inhibitors (SGLT2i) are among the first classes of drugs approved for the treatment of type 2 diabetes mellitus (Bailey and Day, 2019). These agents lower blood sugar by inhibiting the reabsorption of glucose by the kidney (van Bommel et al., 2017) and excreting excess glucose from the urine (DeFronzo et al., 2017). As per the American Diabetes Association (ADA), patients with high-risk factors or those diagnosed with Atherosclerotic Cardiovascular Disease (ASCVD) or heart failure (HF) are advised to select treatments that are beneficial for this population, including SGLT2i (ElSayed et al., 2023). Notably, SGLT2i are also the first hypoglycemic agent shown to reduce the risk of CVD in a cardiovascular outcome study (DeFronzo et al., 2017). In clinical trials, treatment with SGLT2i was observed to reduce the number of patients with type 2 diabetes with high cardiovascular risk (Joseph et al., 2022), as well as reduce the risk of cardiovascular death or hospitalization for HF in these patients (Kansara et al., 2022). Dapagliflozin (Maltês et al., 2021), Canagliflozin (Neal et al., 2017), Empagliflozin (Wanner et al., 2016), or Ertugliflozin (Markham, 2018) are the four SGLT2i approved for marketing in China, out of a total of seven SGLT2is approved worldwide. Several experiments have proved that the improvement of SGLT2i drugs on the prognosis of CVDs, especially HF, is not limited to type 2 diabetes patients, but can be more widely used in clinical broad-spectrum treatment of CVDs (Braunwald, 2022). Current studies have found that SGLT2i can improve myocardial contractility, overall cardiac function, endothelial function, reducing myocardial fibrosis, and so on (Lu et al., 2020).

Bibliometrics is an interdisciplinary science that uses knowledge from mathematics and statistics to analyze knowledge vectors using the above fields quantitatively. As a research type that combines mathematics, statistics and philology, bibliometrics focuses on quantification in the research process and is a comprehensive statistical method of literature and information (Wallin, 2005).

Since the full results of the DAPA-HF trial were officially announced at the ESC2019 Annual Meeting, SGLT2i has not only been considered as an important drug for the treatment of type 2 diabetes but has become a hot research topic in the cardiovascular field as well. Since 2020, the research of SGLT2i in the field of CVD has continued to expand. In this paper, we conducted literature information processing and mathematical statistical analysis on the articles related to the application of SGLT2i in CVDs, evaluated the achievements and impacts of relevant studies in the past 5 years from the perspective of bibliometrics, explored key topics in this field, followed up the latest progress, and looked forward to possible future research directions.

## 2 Methods

### 2.1 Searching strategy and data collection

**FIGURE 1:**
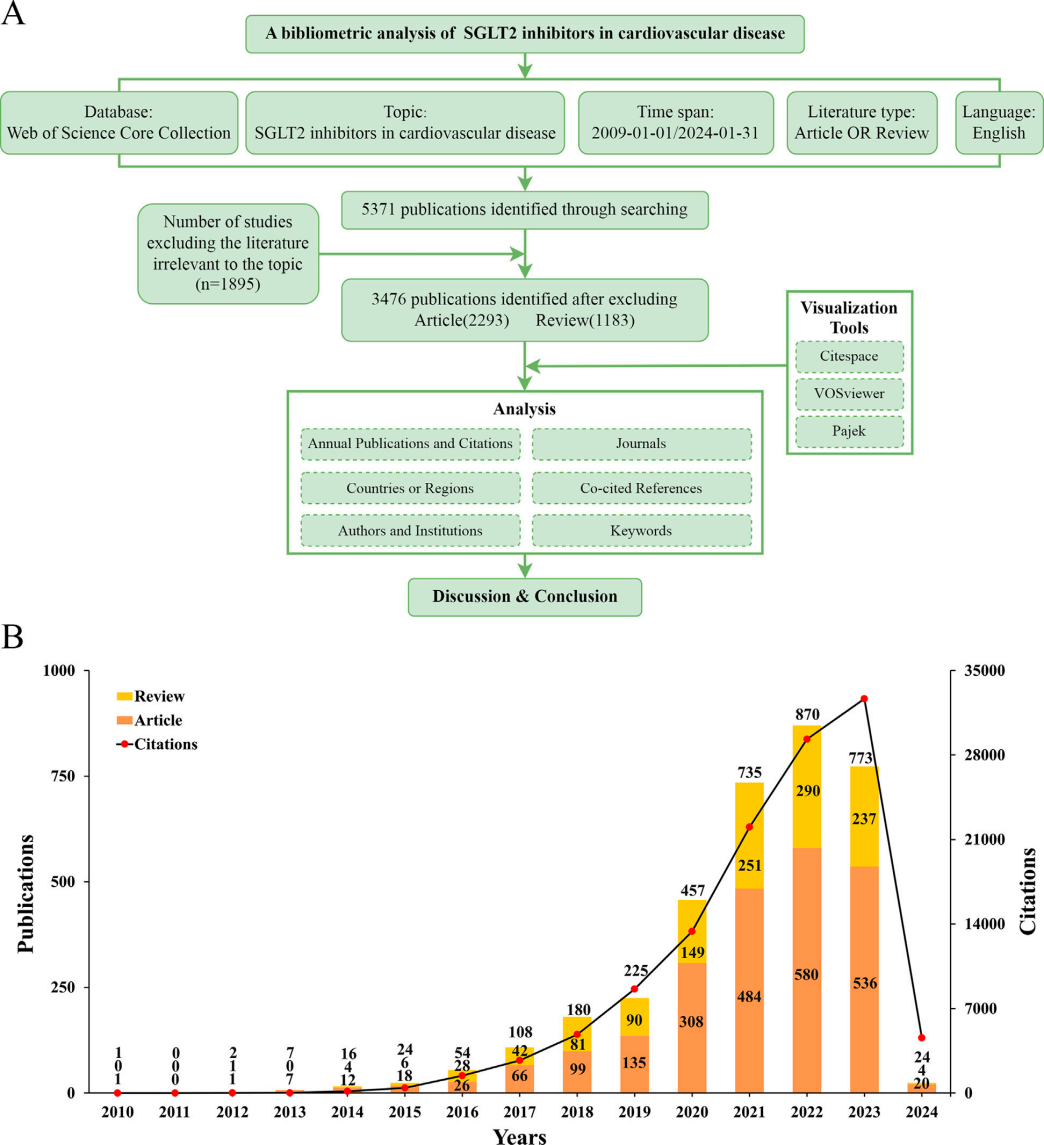
**(A)** Flowchart of bibliometric analysis. **(B)** The number of articles about cardiac transplants for heart failure per year from 2009 to 2023.

The Web of Science database (https://login.webofknowledge.com/) was used to search publications for conducting bibliometric analysis. The search and data collection were accomplished on 18 March 2024. All publications were output through the format of “Full Record and Cited References” in the form of plain text files. Below is the search formula applied. TS = (“SGLT2 inhibit*” OR “sodium–glucose cotransporter 2 inhibit*” OR “sodium–glucose cotransporter type 2 inhibit*” OR “sodium–glucose linked transporter type 2inhibit*” OR “sodium glucose cotransporter 2 inhibit*” OR “sodium/glucose cotransporter 2 inhibit*” OR “sodium–glucose co-transporter 2 inhibit*” OR “sodium–glucose transporter 2 inhibit*” OR “sodium glucose co-transporter 2 inhibit*” OR “sodium–glucose transport protein 2 inhibit*” OR “sodiumdependent glucose cotransporter 2 inhibit*” OR “sodium–glucose contransporter proteins 2 inhibit*” OR “sodium-dependent glucose transporters 2 inhibit*” OR “sodium glucose transport protein 2inhibit*” OR “gliflozin*” OR “canagliflozin*” OR “dapagliflozin*” OR “empagliflozin*” OR “ertugliflozin*” OR “ipragliflozin*” OR “luseogliflozin*” OR “tofogliflozin*”) AND TS = (“high blood pressure” OR hypertensi* OR “peripheral arter*” disease* OR “atrial fibrillat*” OR tachycardi* OR endocardi* OR pericard* OR ischem* OR arrhythmi* OR thrombo* OR cardio* OR cardiac* OR “heart failure” OR “heart beat” OR “heart rate*”OR “heart val*” OR coronary* OR angina* OR ventric* OR myocard*) AND DT = (Article OR Review) AND LA = (English) AND DOP = (2009-01-01/2024-01-31). Two investigators (Melisandre Wan and Yunyi Zhang) searched and filtered the publications. Disagreements were discussed with the corresponding authors until a consensus was reached (Figure 1).

### 2.2 Data standardization

Screen relevant literature keywords according to standards to prevent non-standardized keywords from causing inconsistencies in part-of-speech, plural, and singular numbers in the keyword co-occurrence map, avoid duplication of synonyms, and enhance the rigor of analysis. A standardization of countries or regions was also conducted, such as merging England, Scotland, Wales, and Northern Ireland into the United Kingdom, Taiwan/Hong Kong to China, and Turkey to Turkiye.

### 2.3 Visualized analysis

The software used in our study is as follows: VOSviewer (version 1.6.18, Leiden University, Leiden, Netherlands), CiteSpace (version 6.2.R4, Chaomei Chen, Drexel University, Philadelphia, PA, United States), Microsoft Excel (Redmond, WA, United States) 2019, based on the R language Bibliometrix package (4.1.3 package) and Scimago Graphica (beta version 1.0.36). These applications are used to analyze the data and export the results into tables summarizing bibliometric parameters, including numbers and years of publications; total and average citations; title; country and institution; authors; journal; keywords; and references. The inter-country co-occurrence diagram was generated in VOSviewer and Scimago Graphics, and the inter-institutional co-occurrence collaboration diagram was generated in VOSviewer. As shown in the VOSviewer diagram, nodes corresponding to weights are links indicating collaborative relationships or co-occurrences in a single document, while link thickness indicates a positive correlation with link strength. In the Visualize Networks module, use colors to differentiate clusters. In the overlay visualization module, node color represents the average year of publication. Blue represents previous research phases and yellow represents the current phase. We used CiteSpace to generate keyword burst maps and identify references. Darker and lighter nodes represent earlier and later publications, respectively. Additionally, keywords and references in the burst module are sorted by the year the burst started. The three-domain graph was analyzed using the Bibliometrix package (4.1.3 package) based on R language to display the relationship between co-cited references, authors, and keywords.

## 3 Results

### 3.1 Analysis of the publication and citation trends

According to the search strategy briefly described above, a total of 5,371 publications were retrieved. After removing literature irrelevant to this topic (n = 1,895), 3,476 studies were identified as meeting the scope of our study, including articles (n = 2,293) and reviews (n = 1,183). We found an overall growth trend in publications at the intersection of SGLT2 and CVD globally, from one publication in 2010, peaking at 890 publications in 2022, and falling back to 773 publications in 2023. As of the final target date for our study (31 January 2024), the number of studies published in 2024 was 24. The specific situation is shown in Figure 1A.

Figure 1B showed that in 2010, the first article focusing on this field was published. From 2010 to 2013, relevant publications were always in single digits, and the number of citations was also in single digits to around 20. This phenomenon changed only in 2014 when the number of citations reached more than 100 times for the first time. From 2014 to 2019, the number of publications increased year by year, but it was not significant enough. From 2019 to 2023, research began to grow rapidly and explosively. From 2021 to 2023, the growth rate decreased slightly, and the most significant increase was from 2020 to 2021. The articles obtained for this study were published before 31 January 2024. We speculate that the number of publications in 2024 will equal or exceed the number in 2023. The number of citations is also increasing year by year, showing an upward trend, which also corresponds to the increasing number of publications. The increasing number of articles means that research activities in this field are still attracting attention, and the increasing number of citations means that exploration has not yet reached saturation. All in all, this intersectional field began in 2010, entered a period of rapid growth about 10 years later, and continued to attract the attention of researchers even until today.

### 3.2 Analysis of cooperation status

#### 3.2.1 Countries/regions

**FIGURE 2:**
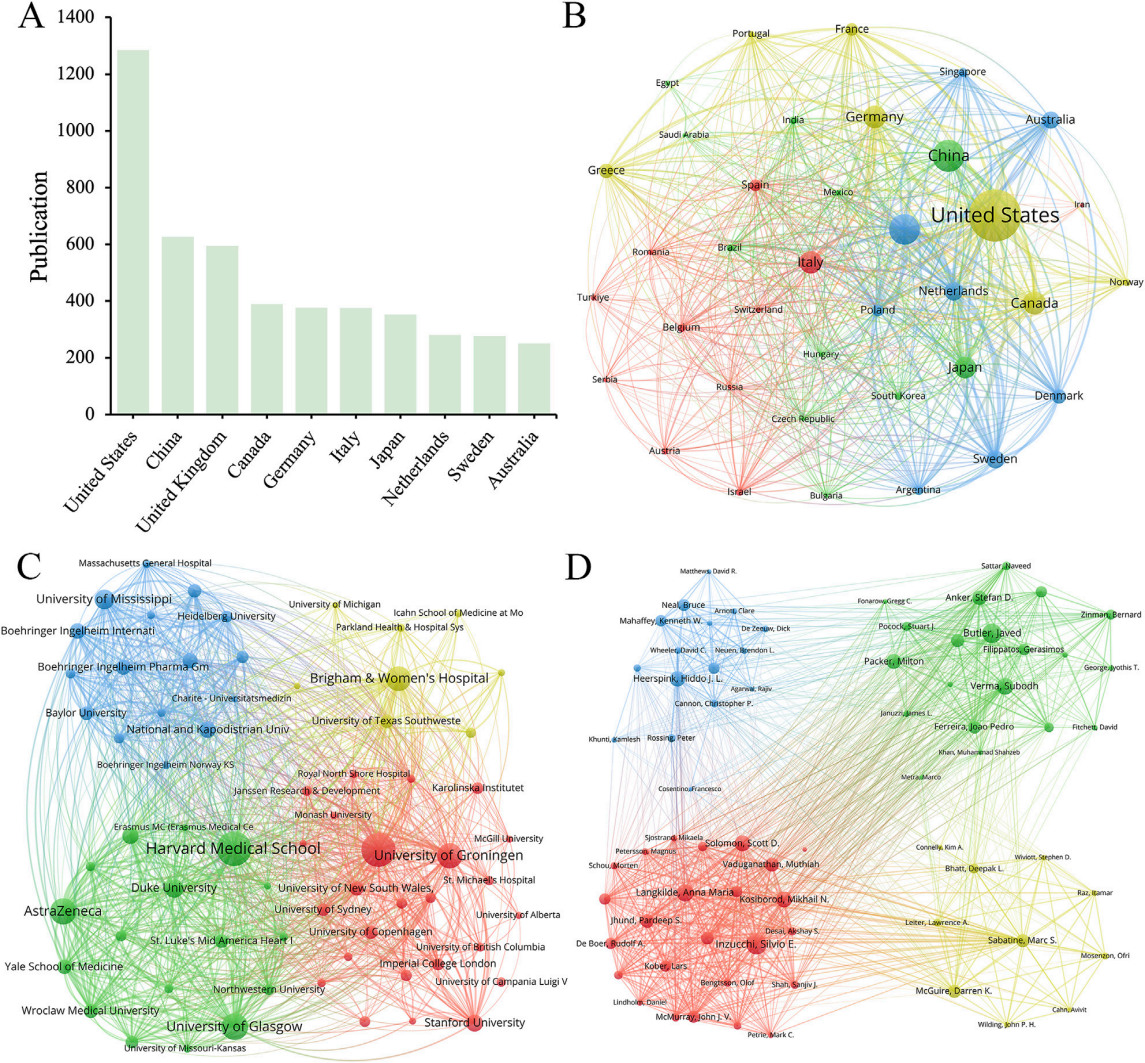
**(A)** The top 10 productive countries/regions. The network maps show countries/regions, **(B)** institutions, **(C)** and authors **(D)** involved in the research on SGLT2 inhibitors in cardiovascular disease.

Figure 2 showed the number of publications and cooperation status of various countries, institutions, and authors who had contributed to SGLT2 in the field of CVDs. A total of 37 countries met the minimum number of publications of 20 or more, among which the United States had the largest number of relevant publications (1,285), accounting for 36.97% of the total. China was the country with the largest number of published documents after the United States (627, 18.04%). The other countries in the top ten (The top 10 productive countries) were the United Kingdom (594, 17.09%), Canada (389, 11.19%), Germany (377, 10.85%), and Italy (376, 10.82%), Japan (353, 10.15%), Netherlands (281, 8.08%), Sweden (276, 7.94%) and Australia (250, 7.19%) (Figures 2A, B). This result demonstrated that the United States (1,285 articles, 73,337 citations, and 3,012 total link strength) played a dominant role in published contributions to SGLT2 in CVD.

#### 3.2.2 Organization

From a total of 4,300 institutions, we filtered according to the criteria of publishing no less than 30 publications, and then clustered the selected 66 cooperative institutions to form a cluster diagram (Figure 2C), which showed the 10 institutions with the highest productivity. The institution that published the largest number of papers was Harvard Medical School (234), followed by the University of Toronto (232), the University of Glasgow (168) and AstraZeneca (166). Harvard Medical School collaborated most closely with other institutions, with a total tie strength of 1,933, followed by the University of Toronto (1,814). It was worth noting that the number of Citations of the University of Toronto was much higher than that of other institutions, reaching 20,595, while the number of Citations of Harvard Medical School, which published the most papers, is 13,763. Among the top 10 institutions by publication volume, five institutions belonged to the United States, and the others were from the United Kingdom (2 institutions), Canada, the Netherlands, and Poland. We found that most of the partner institutions belonged to the same country, and among the top ten, the number one published by Harvard Medical School (234 articles) was more than 2.5 times that of the tenth-ranked University of Missouri (92 articles). This result indicated that relevant research should strengthen cooperation between domestic and transnational institutions.

#### 3.2.3 Author

Figure 2D showed authors whose minimum number of publications was greater than or equal to 20. Within the scope of articles that meet the standards, there were 15,157 authors, and after refinement, 74 authors met the requirements. Among the top 10 productive authors in the field of SGLT2i and CVD, Inzucchi, Silvio E. (111 publications, 4,787 citations) and Butler, Javed (106 publications, 4,465 citations) were the most prolific. Heerspink, Hiddo J. L. was the author with the highest rankings in both Total citation and Per citation. His articles had been cited a total of 5,993 times, and the Per citation reached 74.91, indicating that his published articles had high scientific value (Table 1). Hiddo’s research around SGLT2i mainly included its impact on patients with CVD and chronic kidney disease (Heerspink et al., 2020; Perkovic et al., 2019) (with or without CVD) (Brown et al., 2021). Researchers also paid attention to the DAPA-CKD trial (Wheeler et al., 2021), disease outcomes (van der Aart-van der Beek et al., 2022), etc.

**TABLE 1:** The top 10 productive authors.

Name	Country	Publications	Total citations	Per citations
Inzucchi, Silvio E.	United States	111	4,787	43.13
Butler, Javed	United States	106	4,465	42.12
Langkilde, Anna Maria	Sweden	91	4,020	44.18
Verma, Subodh	Canada	89	4,576	51.42
Kosiborod, Mikhail N.	United States	83	3,045	36.69
Solomon, Scott D.	United States	83	3,394	40.89
Heerspink, Hiddo J. L.	Netherlands	80	5,993	74.91
Packer, Milton	United States	79	4,461	56.47
Anker, Stefan D.	Germany	75	3,786	50.48
Vaduganathan, Muthiah	United States	73	1,781	24.40

### 3.3 Journal analysis

**FIGURE 3:**
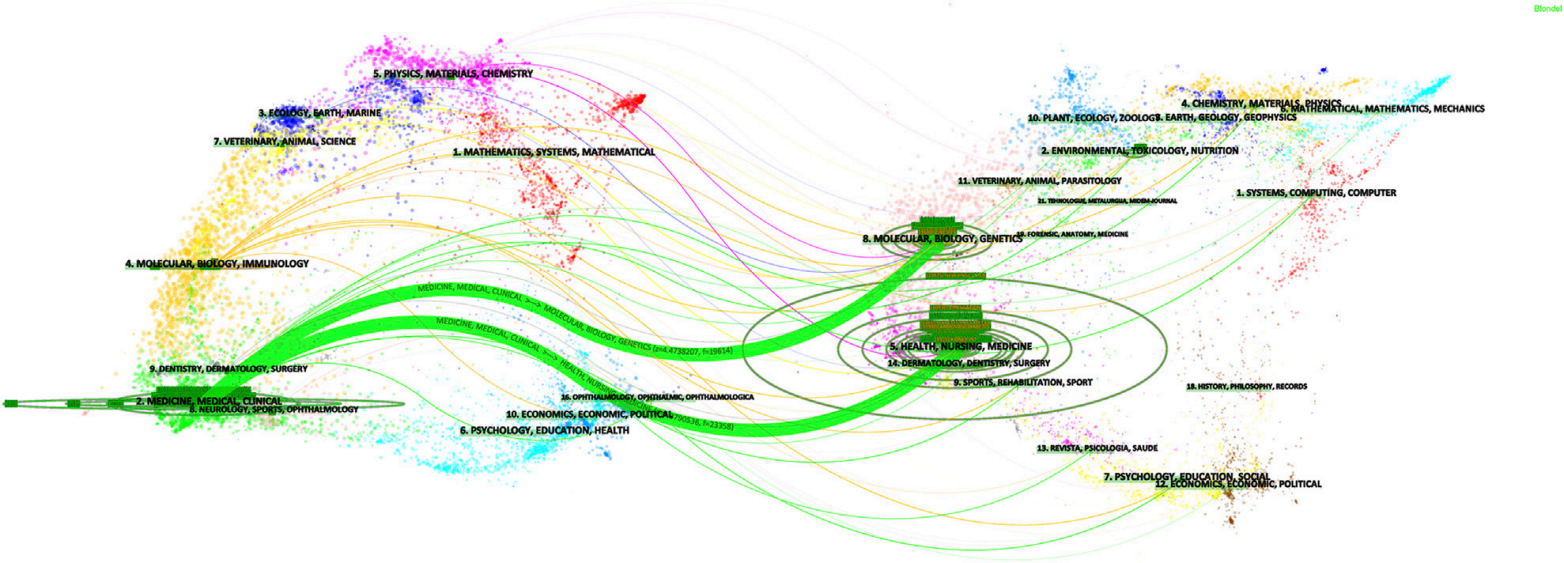
The dual-map overlay of journals.

Table 2 showed the rankings of the 10 journals with the highest publication volume related to the association between SGLT2 and CVD and their co-cited journals. The top three most cited journals were the New England Journal of Medicine (26,120 times), Circulation (11,831 times), and Cardiovascular Diabetology (7,851 times). Figure 3 showed the fields of journal distribution of citing documents and cited documents. The green thick line path marked that the cited journals were mainly from the fields of Medicine, Medical, and Clinical, while the citing journals were mainly from the fields of Health, Biology, Genetics, etc. field.

**TABLE 2:** The top 10 journals of publications on SGLT2 inhibitors in cardiovascular disease (sorted by total citations).

Journal	Category	Impact factor (2022)	Total publications (%)	Total citations
New England Journal of Medicine	Medicine, General and Internal	158.5	14 (0.40)	26,120
Circulation	Cardiac and Cardiovascular Systems; Peripheral Vascular Disease	37.8	77 (2.22)	11,831
Cardiovascular Diabetology	Cardiac and Cardiovascular Systems; Endocrinology and Metabolism	9.3	182 (5.24)	7,851
Diabetes Care	Endocrinology and Metabolism	16.2	56 (1.61)	6,203
Diabetes Obesity and Metabolism	Endocrinology and Metabolism	5.8	163 (4.69)	4,346
European Journal of Heart Failure	Cardiac and Cardiovascular Systems	18.2	83 (2.39)	4,235
Journal of the American College of Cardiology	Cardiac and Cardiovascular Systems	24.4	43 (1.24)	3,850
Lancet	Medicine, General and Internal	168.9	5 (0.14)	3,182
European Heart Journal	Cardiac and Cardiovascular Systems	39.3	44 (1.27)	2,849
Diabetologia	Endocrinology and Metabolism	8.2	26 (0.75)	2,639

### 3.4 Analysis of co-cited references

**FIGURE 4:**
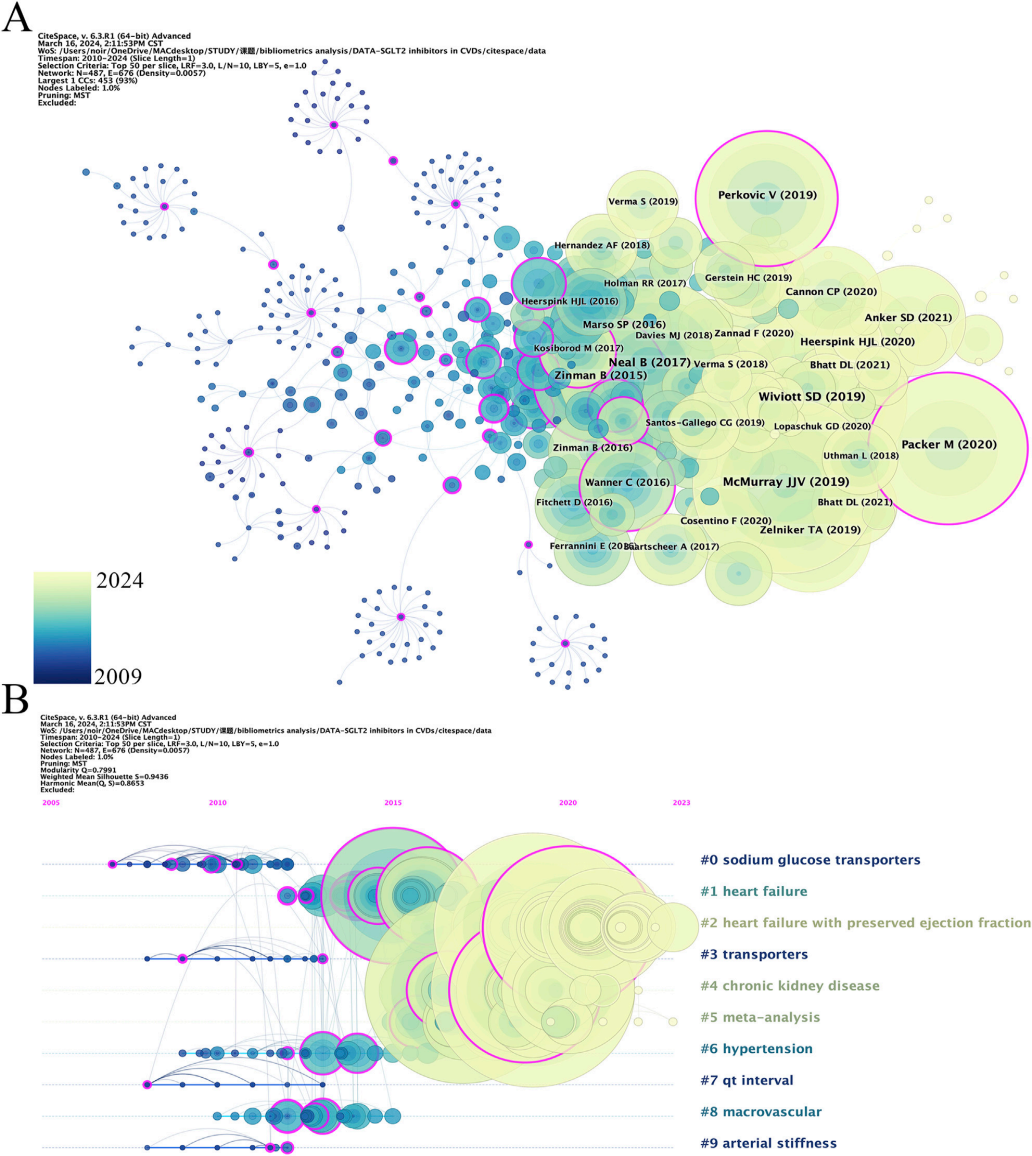
The map of co-citation references **(A)** and the largest 10 clusters **(B)**.

When two documents are cited together by other publications, we call the documents co-cited references. Each node in Figure 4A represents a cited document. The larger the node area, the more citations the document has. Information on the top 10 co-cited references related to SGLT2i is listed in Table 3. The most co-cited document was Canagliflozin and Cardiovascular and Renal Events in Type 2 Diabetes, published by the New England Journal of Medicine in 2017 and Neal as the first author.

**TABLE 3:** The top 10 co-cited references.

First author	Year	Journal	Title	Co-citations
Neal, et al.	2017	New England Journal of Medicine	Canagliflozin and Cardiovascular and Renal Events in Type 2 Diabetes	1,588
Wiviott, et al.	2019	New England Journal of Medicine	Dapagliflozin and Cardiovascular Outcomes in Type 2 Diabetes	1,403
McMurray, et al.	2019	New England Journal of Medicine	Dapagliflozin in Patients with Heart Failure and Reduced Ejection Fraction	1,333
Packer, et al.	2020	New England Journal of Medicine	Cardiovascular and Renal Outcomes with Empagliflozin in Heart Failure	965
Zinman, et al.	2015	New England Journal of Medicine	Empagliflozin, Cardiovascular Outcomes, and Mortality in Type 2 Diabetes	965
Zinman, et al.	2016	New England Journal of Medicine	Empagliflozin, Cardiovascular Outcomes, and Mortality in Type 2 Diabetes	840
Perkovic, et al.	2019	New England Journal of Medicine	Canagliflozin and Renal Outcomes in Type 2 Diabetes and Nephropathy	778
Heerspink, et al.	2020	New England Journal of Medicine	Dapagliflozin in Patients with Chronic Kidney Disease	607
Zelniker, et al.	2019	Lancet	SGLT2 inhibitors for primary and secondary prevention of cardiovascular and renal outcomes in type 2 diabetes: a systematic review and meta-analysis of cardiovascular outcome trials	605
Anker, et al.	2021	New England Journal of Medicine	Empagliflozin in Heart Failure with a Preserved Ejection Fraction	546

**FIGURE 5:**
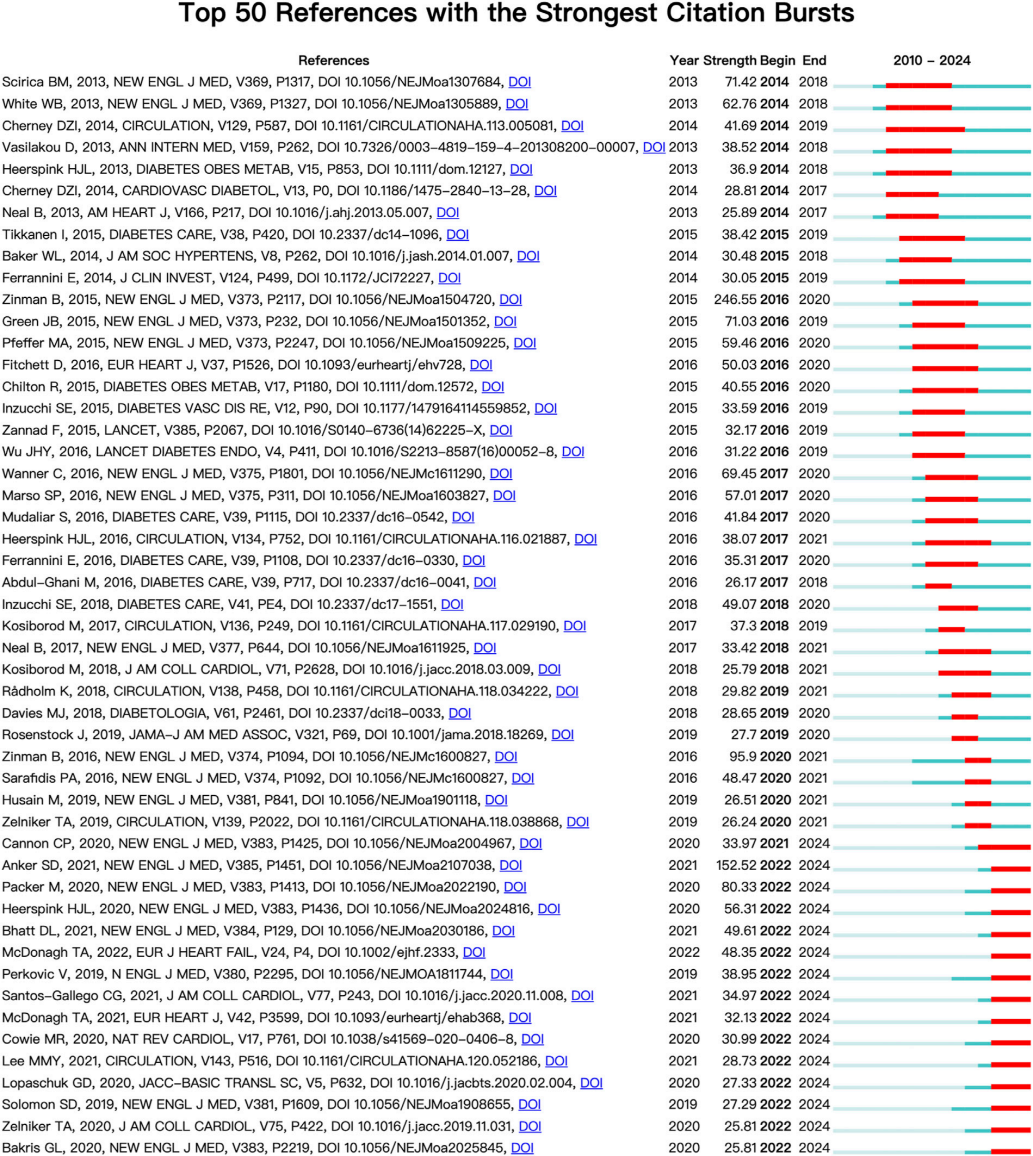
Visual analysis of reference bursts.

Each node in Figure 4 represents a cited document. The size of the graph area displayed by the node on the figure represents the number of times the document has been cited. A node graph generally comprised several layers of circles of different colors, representing situations where the document was noted at different times. Combined with the display of citation burst time in Figure 5, where the red square bar indicated high citation frequency and the blue square bar indicated lower one, we could see the paper entitled *Empagliflozin, Cardiovascular Outcomes, and Mortality in Type 2 Diabetes* published by Zinman B received the strongest burst strength (strength = 246.55, burst period = 2016–2020). References currently in a state of burst are shown in Table 4.

**TABLE 4:** References currently in a state of burst.

Burst period	First author	Year	Journal	Title
2021–2024	Cannon, et al.	2020	New England Journal of Medicine	Cardiovascular Outcomes with Ertugliflozin in Type 2 Diabetes
2022–2024	Anker, et al.	2021	New England Journal of Medicine	Empagliflozin in Heart Failure with a Preserved Ejection Fraction
2022–2024	Packer, et al.	2020	New England Journal of Medicine	Cardiovascular and Renal Outcomes with Empagliflozin in Heart Failure
2022–2024	Heerspink, et al.	2020	New England Journal of Medicine	Dapagliflozin in Patients with Chronic Kidney Disease
2022–2024	Bhatt, et al.	2021	New England Journal of Medicine	Sotagliflozin in Patients with Diabetes and Chronic Kidney Disease
2022–2024	McDonagh, et al.	2022	European Journal of Heart Failure	2021 ESC Guidelines for the diagnosis and treatment of acute and chronic heart failure
2022–2024	Perkovic, et al.	2019	New England Journal of Medicine	Canagliflozin and Renal Outcomes in Type 2 Diabetes and Nephropathy
2022–2024	Santos-Gallego, et al.	2021	Journal of the American College of Cardiology	Randomized Trial of Empagliflozin in Nondiabetic Patients With Heart Failure and Reduced Ejection Fraction
2022–2024	McDonagh, et al.	2021	European Heart Journal	2021 ESC Guidelines for the diagnosis and treatment of acute and chronic heart failure
2022–2024	Cowie, et al.	2020	Nature Reviews Cardiology	SGLT2 inhibitors: mechanisms of cardiovascular benefit beyond glycaemic control
2022–2024	Lee, et al.	2021	Circulation	Effect of Empagliflozin on Left Ventricular Volumes in Patients With Type 2 Diabetes, or Prediabetes, and Heart Failure With Reduced Ejection Fraction (SUGAR-DM-HF)
2022–2024	Lopaschuk, et al.	2020	JACC: Basic to Translational Science	Mechanisms of Cardiovascular Benefits of Sodium Glucose Co-Transporter 2 (SGLT2) Inhibitors
2022–2024	Solomon, et al.	2019	New England Journal of Medicine	Angiotensin-Neprilysin Inhibition in Heart Failure with Preserved Ejection Fraction
2022–2024	Zelniker, et al.	2020	Journal of the American College of Cardiology	Mechanisms of Cardiorenal Effects of Sodium-Glucose Cotransporter 2 Inhibitors: JACC State-of-the-Art Review
2022–2024	Bakris, et al.	2020	New England Journal of Medicine	Effect of Finerenone on Chronic Kidney Disease Outcomes in Type 2 Diabetes

### 3.5 Analysis of keywords

**FIGURE 6:**
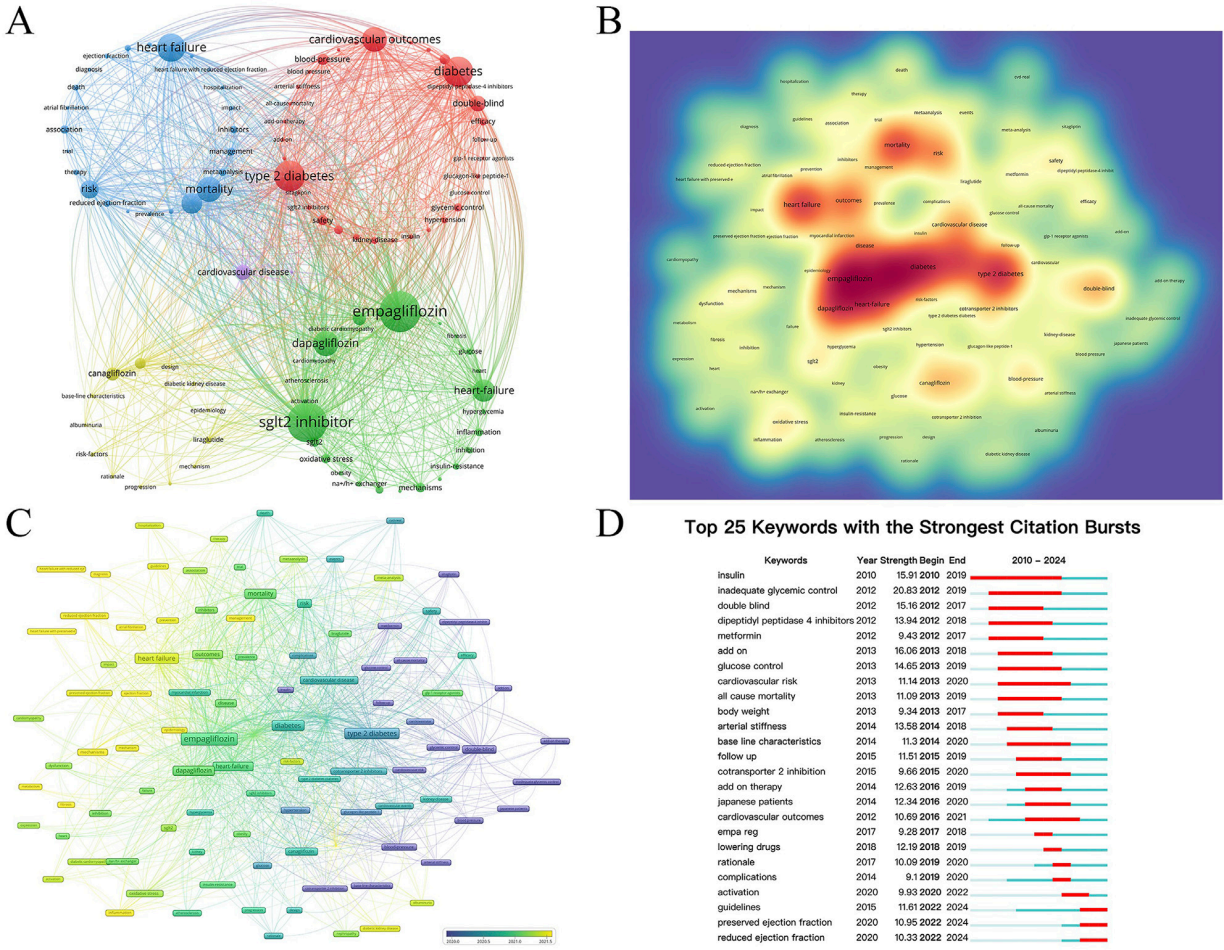
**(A)** The network map of the top 100 keywords with 5 clusters. **(B)** The density view and **(C)** the time view of the map of keywords. **(D)** The keywords with strong citation bursts in articles related to SGLT2 inhibitors in cardiovascular disease.

Figure 6 analyzes the keywords reflecting the hot spots in the intersection of SGLT2i and CVD. A total of 6,632 keywords appeared within the statistical scope, and the top 100 keywords with the highest frequency of occurrence (keywords with a minimum number of posts greater than or equal to 55) were refined for clustering (Figure 6A). Figure 6B is a density map. The darker the red, the higher the probability that this keyword is mentioned. It could be seen from the figure that Empagliflozin, Dapagliflozin, diabetes, and heart failure were the keywords that most frequently appeared in this cross-field, suggesting that there were relatively many studies on Empagliflozin and Dapagliflozin in SGLT2i, and their impacts on HF in CVD were also receiving the most attention.

## 4 Discussion

Our study used bibliometric analysis methods to summarize the latest progress in related fields, identify research hotspots, and most importantly, discuss the current development and future development predictions of the application of SGLT2i in CVDs. We aim to conduct a comprehensive bibliometric analysis of research progress in this field using the Bibliometrix R software package. Through visual charts and graphs as well as other statistical data of countries and regions, institutions, authors, references, and keywords in the field, combined with bibliometric analysis, valuable research results can be provided to grasp the research hot spots and trends in this field.

### 4.1 Bibliometric information

Since phlorizin was isolated from the root bark of the apple tree in 1835 (Ehrenkranz et al., 2005), research on this compound has continued. As phlorizin non-selectively inhibits SGLT1 and SGLT2 and is easily hydrolyzed by β-glucosidase in the small intestine, having low bioavailability, it has not been used in the clinical treatment of diabetes (Martus et al., 2023; Blaschek, 2017). Researchers keep modifying phlorizin and have developed an SGLT2 inhibitor that can be used clinically. In 2010, the results of phase III clinical trial of SGLT2i were awaited and would determine whether the risk-benefit ratio allowed approval of this new drug for the treatment of type 2 diabetes (Nair and Wilding, 2010); in 2012, Dapagliflozin, the first SGLT2i was approved in Europe; in 2016, the REFORM trial was launched to test the safety and efficacy of Dapagliflozin in diabetic patients with known CVD (Singh et al., 2016).

SGLT2i was initially investigated in 2015 for cardiovascular illness (Verma et al., 2018), and attracted widespread attention after the DAPA-HF study published in 2019 (McMurray et al., 2019). Since then, the study has progressively gained prominence and developed into a center for research. Figure 1B illustrated that in 2022, the total number of linked publications reached a maximum of 870 articles; in 2023, the total number of citations reached a maximum of 32,653 times. One possible explanation for the small decline in published publications in 2023 could be the COVID-19 pandemic’s worldwide effects (Weiner et al., 2020). Nonetheless, the number of citations has not decreased in 2023, indicating the continued popularity of research on SGLT2i in cardiovascular disorders. Articles published after January 2024 are not covered due to the deadline for collection; therefore, the curve shown in Figure 1B reaches its peak in 2023, linking to the increasing numbers of articles year by year.

For international cooperation, as shown in Figure 2A, the United States has the largest number of relevant publications (1,285), accounting for 36.97% of the total. Among the top 10 institutions by publication volume, five institutions belong to the United States, and the number of Citations of the University of Toronto is much higher than that of other institutions. These data suggest that the United States is far ahead of other countries in international cooperation, reflecting the United States’ leading and core position in this field. These findings also imply that the University of Toronto as an organization has produced excellent work in this area and deserves interchange of other researchers. China has the second-highest publishing volume after the United States, but its institutional strength and the quantity of citations in its papers still need to be strengthened. Possible reasons include that China’s research in related fields started late and slowly, and there may be a certain number of papers published in Chinese in the academic circle, which are not included in the statistics.

The total number of publications in China reached 627, ranking second among the 90 countries in the statistical scope, second only to the United States (Figure 2A). However, the number of citations to published documents in China stands at only 22,474, and the total link strength is only 857, surpassing only Italy, which ranks sixth in publication volume (n = 376). Among the results of the top 10 productive institutions shown in Table 5, no Chinese institution is among them. From this result, it can be found that although there are a large number of papers related to the intersection of SGLT2i and CVD fields published in China, the influence of Chinese scientific institutions focusing on this area of research is not large enough yet. This may be due to the insufficient exploration of basic research on molecular mechanisms in China, suggesting that the quality of China’s research on SGLT2i and CVD still needs to be improved. However, as international cooperation grows, China will be able to raise the caliber of its research in this field. Harvard Medical School (US), the University of Toronto (Canada), the University of Glasgow (UK), AstraZeneca (UK), University of Groningen (Netherlands), Brigham and Women’s Hospital (US), University of Mississippi (US) and other institutions all made influential contributions to this field of research, which are cooperative partners Chinese institutions can take into consideration.

**TABLE 5:** The top 10 productive institutions.

Institution	Publications	Citations
Harvard Medical School	234	13,763
University of Toronto	232	20,595
University of Glasgow	168	9,933
AstraZeneca	166	9,993
University of Groningen	157	16,061
Brigham and Women’s Hospital	153	13,300
University of Mississippi	115	4,891
Duke University	107	6,538
Stanford University	95	10,972
University of Missouri	92	4,535

Overall, the bibliometric results show that research on SGLT2i in heart disease has continued to develop rapidly in the past 5 years, as the number of documents and the universality of the application of SGLT2i have increased at a higher rate. Researchers from various countries have contributed to different aspects of this field, collectively fostering opportunities for both current and future academic advancements and pharmaceutical innovations.

### 4.2 Detection of research hotspots and future directions

#### 4.2.1 Molecular mechanisms of the role by SGLT2i

Studies have shown that even in non-diabetic individuals (Seman et al., 2013), SGLT2i causes glycosuria and natriuresis while increasing glucagon and ketones (Heise et al., 2013). In other words, the metabolism of SGLT2i drugs in diabetic patients and non-diabetic patients is the same (Al-Jobori et al., 2017).

Numerous studies have observed the effects of SGLT2i on kidney-related functions and their cardiovascular benefits (Lambers Heerspink et al., 2013). Empagliflozin and dapagliflozin can enhance the angiogenesis ability of diabetic EPCs (Neal et al., 2017), and volume contraction was a key determinant of the benefit observed in the trial. It was concluded that the key factor in reducing CV death with SGLT2i is that empagliflozin reduces plasma volume through its diuretic and more conducive properties of glucose and sodium excretion through the glomerulus (Lambers Heerspink et al., 2013), thereby reducing vessel wall load while reducing the risk of sudden cardiac decompensation (Inzucchi et al., 2018).

The hemodynamic changes in empagliflozin-treated patients may be accompanied by increased erythropoiesis. This is because erythropoietin-induced increases in hematocrit may be caused by direct enhancement of myocardial and systemic tissue oxygen delivery, which may improve myocardial function (Ekanayake and Mudaliar, 2023). One study found that under long-term SGLT2i intervention, plasma erythropoietin concentration increased by 31 (64) % (*p* = 0.0078) (Ferrannini et al., 2017). Another study in 2020 proposed that an increase in hematocrit was consistently observed in all SGLT2i studies. It was believed that the phenomenon was at least partly due to SGLT2i reducing glomerular filtration rate (especially the effect on blood flow, and filtration rate of sodium), stimulating increased erythropoietin levels and promoting red blood cell production (Packer, 2023). Beta-hydroxybutyrate is also thought to help stimulate the production of erythropoietin possibly. A 2023 study suggested that the liver might play a role in erythropoietin production during SGLT2 inhibition. It proposed that hypoxia-inducible factor-2α (HIF-2α) and erythropoietin are co-expressed in hepatocytes, facilitating erythropoietin synthesis in the liver by promoting the transcription of the erythropoietin gene and upregulating SIRT1. The increase in SIRT1 also exerts direct cytoprotective effects on the heart (Packer, 2023; Packer, 2024). These series of studies continue to reveal in depth the mechanism by which SGLT2i affects erythropoiesis and cardio protection and enhances our understanding of the cellular mechanisms by which SGLT2i improves HF outcomes in clinical trials. Additionally, research has suggested that the application priority of SGLT2i in ASCVD is going to be greater than in HF (Koh, 2024).

In addition to volume changes, SGLT2i may optimize loading conditions by lowering blood pressure and altering vascular function. A recent study showed that empagliflozin reduced central and 24-hour systolic and diastolic blood pressure, central pulse pressure, and forward wave amplitude in patients with type 2 diabetes (Striepe et al., 2017). Other studies have shown that SGLT2i improves endothelial function and aortic stiffness index (Chilton et al., 2015) and may induce vasodilation through activation of voltage-gated potassium (Kv) channels and protein kinase G (Solini et al., 2017; Li et al., 2018).

Cancer and CVD are the two leading causes of death worldwide (Naghshi et al., 2021). Cardiotoxicity is a critical and common adverse effect of cancer-related chemotherapy (Herrmann, 2020; Kourek et al., 2022). Chemotherapy-induced cardiotoxicity is associated with various cancer treatments, such as anthracyclines, immune checkpoint inhibitors, and kinase inhibitors (Asnani and Peterson, 2017). Various types of SGLT2i can reduce the cardiotoxicity of this type of anti-cancer drug in humans (Vaziri et al., 2023). Empagliflozin has a slowing effect on cardiotoxicity caused by commonly used chemotherapy drugs 5-fluorouracil (5-FU) (Refaie et al., 2024), doxorubicin (Barış et al., 2021; Malik et al., 2024), trastuzumab (Min et al., 2023) and sunitinib (Ren et al., 2021), and can prevent QT interval prolongation caused by amitriptyline (Belen et al., 2022). Empagliflozin can also improve myocardial strain, reduce cardiac fibrosis and pro-inflammatory cytokines in non-diabetic mice treated with doxorubicin (Lee et al., 2017), and improve diabetic cardiomyopathy (Zhang et al., 2023). Canagliflozin is considered a potential cardioprotective drug, but it does not significantly alleviate the cardiotoxicity induced by pirarubicin (Shi et al., 2021); dapagliflozin has a good effect in relieving or preventing animal cardiotoxicity caused by doxorubicin (Hsieh et al., 2022; Chang et al., 2022; Chang et al., 2021; Mahmoud Refaie et al., 2023), cadmium (Refaie et al., 2022), and cyclophosphamide (Quagliariello et al., 2021; Avagimyan et al., 2023).

Recently, the molecular mechanisms underlying the cardiovascular protective effects of SGLT2i are still being explored. Dapagliflozin can mediate M2 polarization via the STAT3 pathway, thereby reducing myofibroblast infiltration during post-infarction remodeling (Lee et al., 2017). A study newly published in August 2024 focused on how dapagliflozin mitigates myocardial cell injury induced by acute myocardial infarction by increasing the sirtuin family (SIRT1/SIRT3) and cascade signaling, finding that dapagliflozin increases the gene and protein expression of SIRT1, SIRT3, and SIRT6, and antagonizes the hypoxia-induced downregulation of genes such as ESRRA, EPAS1, and AGTRAP (Lin et al., 2024). An earlier study in 2024 also noted that canagliflozin alleviates cardiac hypertrophy in mice caused by high salt intake, which suppresses SIRT3 expression in the heart (Zhao et al., 2024). In addition to the Sirtuin family, the NF-κB-related inflammatory cascade is another important mechanism of action for SGLT2 inhibitors. Dapagliflozin can prevent the reduction of ejection fraction and decrease myocardial and renal NF-κB expression, thereby providing various cardiometabolic benefits in clinical settings (Quagliariello et al., 2024). Similarly, empagliflozin downregulates NF-κB expression and mitigates cardiotoxicity induced by other drugs via the TNFα/TLR/NF-κB signaling pathway (Refaie et al., 2024).

The effect of SGLT2i on improving cardiovascular health in patients with CVD, with or without type 2 diabetes mellitus, is a combination of multiple mechanisms, including promoting vascular endothelial regeneration and enhancing liver erythropoietin production function and renal filtration. Studies also have delved into the mechanisms at the genetic and signaling cascade levels, exploring the effects of various types of SGLT2i on the STAT3 pathway, the sirtuin family, and the NF-κB-related inflammatory cascade. The function and mechanism of SGLT2i will continue to be the attention of researchers.

#### 4.2.2 Applications of SGLT2i in CVDs

Researches find that the most obvious effect of SGLT2i drugs in clinical application is the substantial reduction of HHF in patients with diabetes (Chen et al., 2023). In addition to the main role in treating diabetes, researchers have begun to focus on the function of SGLT2i in the treatment of HF (Nikolic et al., 2022).

In 2015, a study conducted the EMPA-REG OUTCOME (Empagliflozin Treatment of Type 2 Diabetes Cardiovascular Outcomes Trial) trial including 7,020 subjects with type 2 diabetes and found that SGLT2i can cause CV death, finding that nonfatal MI or nonfatal stroke was reduced by 14%, and the relative risk of CV mortality was reduced by 38%, while no significant effect was on atherosclerotic ischemic events (Zinman et al., 2015). The CANVAS project (Canagliflozin Cardiovascular Assessment Study) found that SGLT2i did not significantly reduce the risk of CV death in patients, but the relative risk of HHF was significantly reduced (33%). This project also confirmed that the cardiovascular outcome most significantly affected by SGLT2i intervention was HF (Neal et al., 2017). The DECLARE-TIMI 58 (Effects of Dapagliflozin on Cardiovascular Events) project found that treatment with SGLT2i drugs was associated with a 17% reduction in CV death or HHF outcomes (Wiviott et al., 2019).

In recent years, more studies have noted that the effect of SGLT-2i on CVD is independent of its effect on lowering blood glucose (Striepe et al., 2017), in other words, extending the therapeutic benefits of SGLT2i to non-diabetic patients (Bonora et al., 2020). In both DAPA-HF and EMPEROR Reduced trials, SGLT2i was found to reduce the risk of the primary composite endpoint of cardiovascular death or HF hospitalization (Zannad et al., 2020).

In addition to HF, researches on the impact of SGLT2i on other CVDs such as atherosclerosis, coronary heart disease (Alatawi, 2024), cerebrovascular disease, rheumatic heart disease, and congenital heart disease (Neijenhuis et al., 2023; Konduri et al., 2023) are also popular. Various SGLT2is have good preventive effects on atrial fibrillation, especially empagliflozin (Zhang et al., 2024). Empagliflozin improves the therapeutic effect of myocardial infarction (Peikert and Vaduganathan, 2024; Aziz et al., 2024; Carberry et al., 2024; Santos-Gallego et al., 2023), and promotes functional recovery after stroke (Desai et al., 2022). Endothelial progenitor cells (EPCs) play an important role in an important endogenous repair mechanism for diabetic vascular complications, while empagliflozin and dapagliflozin can enhance the angiogenesis ability of diabetic EPCs (Vercalsteren et al., 2024; Luo et al., 2023). Both dapagliflozin and canagliflozin can treat gout (Banerjee et al., 2023).

Although its clinical safety has been relatively well-tested. Studies reported different adverse effects of SGLT2i in clinical applications (Mascolo et al., 2022), like hypoglycemia, hypotension, lower limb amputation (Zinman et al., 2015), fractures, genitourinary infections (Wiviott et al., 2019), and diabetic ketoacidosis of varying frequency. But the probability of them is very low, and the occurrence of serious side effects is even less (Neal et al., 2017). Therefore, in those effective experiments on the connection between SGLT2i and CVD, the number of clinical studies and analyses is also extensive. In addition, the clinical exploration of the indications of SGLT2i in different CVDs is also a focus of future research (Table 6).

**TABLE 6:** The top 10 most cited articles.

Title	First author	Journal	Year	Total citations	Citations per year
Canagliflozin and Cardiovascular and Renal Events in Type 2 Diabetes	Neal, et al.	New England Journal of Medicine	2017	4,946	618.25
Empagliflozin, Cardiovascular Outcomes, and Mortality in Type 2 Diabetes	Zinman, et al.	New England Journal of Medicine	2015	4,580	458.00
Dapagliflozin and Cardiovascular Outcomes in Type 2 Diabetes	Wiviott, et al.	New England Journal of Medicine	2019	3,610	601.67
Dapagliflozin in Patients with Heart Failure and Reduced Ejection Fraction	McMurray, et al.	New England Journal of Medicine	2019	3,531	588.50
Management of Hyperglycemia in Type 2 Diabetes, 2018. A Consensus Report by the American Diabetes Association (ADA) and the European Association for the Study of Diabetes (EASD)	Davies, et al.	Diabetes Care	2018	2,458	351.14
Cardiovascular and Renal Outcomes with Empagliflozin in Heart Failure	Packer, et al.	New England Journal of Medicine	2020	2,445	489.00
Dapagliflozin in Patients with Chronic Kidney Disease	Heerspink, et al.	New England Journal of Medicine	2020	2,143	428.60
Empagliflozin in Heart Failure with a Preserved Ejection Fraction	Anker, et al.	New England Journal of Medicine	2021	1,798	449.50
SGLT2 inhibitors for primary and secondary prevention of cardiovascular and renal outcomes in type 2 diabetes: a systematic review and meta-analysis of cardiovascular outcome trials	Zelniker, et al.	Lancet	2019	1,704	284.00
2022 AHA/ACC/HFSA Guideline for the Management of Heart Failure: A Report of the American College of Cardiology/American Heart Association Joint Committee on Clinical Practice Guidelines	Heidenreich, et al.	Circulation	2022	1,359	453.00

### 4.3 Limitation

In this study, we collected, screened, and analyzed publication data from *The Web of Science* database through a scientific search strategy determined in advance, thus obtaining certain reliable results. However, we cannot completely rule out certain limitations in the research process. For example, in the beginning, although the search algorithm used as a method is comprehensive enough, it still may not completely cover all the literature content, causing our research results to be missed in the first step of searching relevant literature. Secondly, the reliance on data from a single database may limit the generalizability of the findings, potentially leading to discrepancies between the results of this study and those of similar studies conducted both domestically and internationally. Furthermore, due to the time limit of searching the literature, inevitably, some recently published articles were not included in the scope of our study.

## 5 Conclusion and perspective

SGLT2i has been proven to have good cardiovascular intervention effects in non-diabetic patients (Balan et al., 2021; Chen et al., 2022; Hernandez et al., 2023). The effects of SGLT2i on HF are most significant, while patients’ cardiovascular and all-cause mortality can also be reduced.

In diabetic and non-diabetic subjects, the efficacy of SGLT2i drugs was almost the same. SGLT2 may also be used alone as an additional medication independent of metformin in patients with established ASCVD (Fu et al., 2024). Other mechanisms of SGLT2i besides hypoglycemic effects indicate that it is likely to be found to be effective against a wider range of clinical symptoms in a wider patient population in the future.

It can be seen that with the deepening of relevant research, the application scope of SGLT2i in CVDs continues to expand, and the application of SGLT2i is a continuing research hotspot. However, there is currently a lack of relevant clinical research on SGLT2i in non-diabetic patients. Therefore, a larger sample size and more researchers will be needed to conduct some studies targeting non-diabetic patients. This type of research will be highly groundbreaking and has relatively complete mechanism support. The rich mechanism of SGLT2i and the data in Tables 3, 4 may also suggest that the exploration of the molecular mechanism of the influence of SGLT2i in CVDs has been a hot research direction in recent years, and breakthroughs have been made continuously in 2020–2023 and may be in the peak trend of continuous improvement and progress.

## Data Availability

The original contributions presented in the study are included in the article/supplementary material, further inquiries can be directed to the corresponding authors.
